# A statistical analysis of the correlations among various types of clinical indexes for patients with chronic hepatitis B: A hospital-based study

**DOI:** 10.1097/MD.0000000000019201

**Published:** 2020-02-21

**Authors:** Jie Wang, Yonglan Pu, Yinhua Gong, Zhonghua Li, Xiaofang Zhu

**Affiliations:** Taicang Affiliated Hospital of Soochow University, The First People's Hospital of Taicang, Suzhou, Jiangsu Province, China.

**Keywords:** CAP, chronic hepatitis B patients, clinical indexes, LSM, statistical analysis

## Abstract

Hepatitis B virus (HBV) is one of the commonest chronic infections, especially in Asia and Africa, which put a heavy burden worldwide. With the advanced knowledge of HBV, early detection, primary care, and hepatology have made huge progression than before. However, the relationship between gender, age, and different key parameters in HBV patients remains to be determined.

In this study, we measured various physiological and biochemical indexes in a large cohort of HBV patients as well as healthy control. We investigated the strength of correlations among those indexes and reported instantaneous imaging results. Moreover, we examined the effects of various grouping modes such as by gender or age on liver stiffness measurement (LSM) and controlled attenuation parameters (CAPs). We compared their diagnostic values for hepatic fibrosis in HBV patients.

The results showed that specimens from a healthy control were obviously clustering tightly together, while the specimens from the HBV patients were clustering into several subgroups. Direct bilirubin (DB), total bilirubin (TB), aspartate aminotransferase (AST), and alanine aminotransferase (ALT) occurred together with the diagnosis of HBV. Furthermore, groups categorized by Gender had significant effects on fibrotouch measurement not only in HBV patients but also in healthy control.

Our research was to evaluate the actual effects of various parameters on Fibrotouch and make improvement of the critical value of those medical indexes.

## 1 Introduction

Chronic hepatitis B (CHB) is one of the most common liver diseases worldwide. Annually, ∼200 million people are diagnosed with CHB,^[1]^ which might lead to cirrhosis. The previous study suggested that liver cirrhosis, particularly hepatitis B virus (HBV)-related cirrhosis, may gradually develop into hepatic carcinoma.^[2]^ Grading and staging of hepatic fibrosis play an important role in diagnosis, treatment, and prognosis in CHB.^[3]^ Statistical analysis as well as evaluation of a large cohort of multiple diagnostic parameters within HBV patients theoretically provided the foundation for routine screening.

Up to date, liver biopsy remains to be the gold standard for evaluating the level of hepatic fibrosis.^[4]^ However, biopsy carries a high risk of trauma and other hazards with a mortality risk of 0.2%.^[5]^ Considering the uneven sampling for liver biopsy and the mostly dependence of subjective experience in pathological interpretation,^[6]^ it is important to employ a simple, non-invasive and highly specific method for measuring hepatic fibrosis.

Non-invasive hepatic fibrosis measurement methods consist of four types:

1. serum fibrosis indices such as hyaluronic acid/IV collagenous fiber;
2. serum index models such as APRI/AARI/FIB-4;
3. imaging methods such as ultrasound, CT and nuclear magnetic resonance; and
4. instantaneous elasticity imaging.

The transient elastography has been widely used to validate the liver stiffness measurement (LSM) and to evaluate the degree of hepatic fibrosis, which is a kind of simple, noninvasive, routine quantitative measurement method. The noninvasive quantitation of liver stiffness by ultrasound has revolutionized the diagnosis of liver cirrhosis. Liver stiffness is an excellent surrogate marker of advanced fibrosis and cirrhosis outscoring all previous noninvasive approaches to detect cirrhosis. Like other soft tissue stiffness, liver stiffness depends on many factors, including the extracellular matrix of the organ, the internal pressure inside the organ, and viscous effects.^[7]^ Hence, how the various parameters of the HBV patients affect the LSM value remains to be fully determined. Fat could affect ultrasound propagation and in order to detect and quantify steatosis, a parameter called controlled attenuation parameter (CAP) has been developed, which is based on the ultrasonic properties of the radiofrequency backpropagated signals acquired. CAP is commonly used as an estimate of the ultrasonic attenuation and acts as a valid tool for the diagnosis of steatosis.

Fibroscan (Echosens, Paris, France) is widely used in clinical practice. It measures the degree of hepatic fibrosis by the instantaneous shear wave technique.^[8–11]^ Studies demonstrated that Fibroscan has high sensitivity and specificity. A comparison with the liver biopsy result found that Fibroscan perfectly distinguished levels of hepatic fibrosis in high consistency.^[12]^ The instantaneous liver elasticity detector Fibrotouch (FT-C, Wuxi Hisky Medical Technologies Co., Ltd.) independently developed in China is basically the same in terms of principle and diagnostic clinical value as Fibroscan.^[13–16]^ The difference is that Fibrotouch measures instantaneous elasticity based on two-dimensional ultrasonic positioning.^[14]^

We conducted a statistical analysis of clinical data in a large cohort of CHB patients in order to understand the role of Fibrotouch in diagnosing hepatic fibrosis. Specifically, we investigated the relationship between the LSM and CAP with gender, age, and other clinical indexes. Our aim was to evaluate the actual effects of various parameters on Fibrotouch and make improvement of the critical value of those medical indexes.

## 2 Methods

### 2.1 Study population

From January 2015 to December 2017, we gathered clinical data from total 1,347 patients including 935 males (69%) and 412 females (31%), who were diagnosed with CHB at the inpatient department of the first People's Hospital of Taicang. Patients, who were HBsAg-positive for 6 consecutive months or had chronic HBV diagnosed on the basis of hepatic histology. The exclusion criteria included superinfection with A, C, D, E hepatitis viruses; cytomegalovirus infection; human herpes virus infection; HIV infection; drug-induced liver injury; hepatocellular carcinoma; the history of partial hepatectomy; biliary tract disease; pregnancy and lactation; and absence of informed consent. We also added 200 healthy participants including 138 males (69%) and 62 females (31%), who have not received any medical treatment or taken medicines more than 6 months as a control to detect the difference between the HBV patients and healthy people. This study was approved by the ethics committee of the first People's Hospital of Taicang, and all participants signed informed consent forms in this study.

### 2.2 Biological parameters

All subjects were asked about their detailed medical records. Meanwhile, general data for all subjects, including gender, age, height, body weight, blood pressure, blood glucose blood lipids were recorded. Indexes including liver functions, hepatitis B serology and viral markers during the same period were also detected and recorded. The biochemical laboratory of the first People's Hospital of Taicang was responsible for serum biochemical analysis and measurement of all subjects recorded, including total bilirubin (TB), direct bilirubin (DB), ALT, and AST. The physicians in the Ultrasound and the Radiology Departments of the first People's Hospital of Taicang conducted abdominal color ultrasonography and MRT measurements to characterize liver morphology and to exclude patients with hepatocellular carcinoma and biliary tract diseases.

### 2.3 Transient electrography

The Fibrotouch (FT-C, Wuxi Hisky Medical Technologies Co., Ltd.) was operated by a professionally trained technician (with >1000 flow processes and a certificate of professional operation) according to the standard of instruments. CAP and LSM measurements were expressed as the median value of at least 10 successful measurements. CAP measurement was calculated simultaneously with the LSM and was only regarded as reliable if successful LSM were obtained.^[17]^

### 2.4 Statistical analysis

Results of APRI, AARI, and FIB-4 were computed according to the following formulae: ARPI (ARPI = AST/(PLT∗100)),^[18]^ AARI (AARI = AST/ALT)^[19]^ and FIB-4 (FIB-4 = Age∗AST/(PLT∗√ALT)).^[20]^ The software package SPSS 22.0 was used for statistical analysis of all 14 indexes.

### 2.5 Correlation analysis

The relationship between each factor was subjected to one-way ANOVA. Enumeration data was subjected to the χ^2^ test. The Spearman analysis was used for correlation testing. Generally, the correlation coefficient 0.8 ≤ |*r*| ≤ 1 indicated that the variables had an extremely strong correlation (*P* ≤ .05). Coefficients of 0.6 ≤ |*r*| < 0.8 indicated that the variables had a strong correlation. Coefficients of 0.4 ≤ |*r*| < 0.6 indicated that the variables had a moderate correlation. Values of |*r*| < 0.4 indicated that the variables had a weak or absent correlation. Measurement data were expressed as mean ± standard deviation. The differences were considered statistically significant when *P* < .05.

### 2.6 Principal components analysis

Principal components analysis (PCA) was commonly used to find patterns in data of high dimension, which was an unsupervised method and would find the greatest sources of variation regardless of the data structure. By using PCA, we could summarize the systematic patterns of variations in our dataset. We used R packages including prcomp and princomp functions.

## 3 Results

### 3.1 Characteristics of the study population

**Figure 1:**
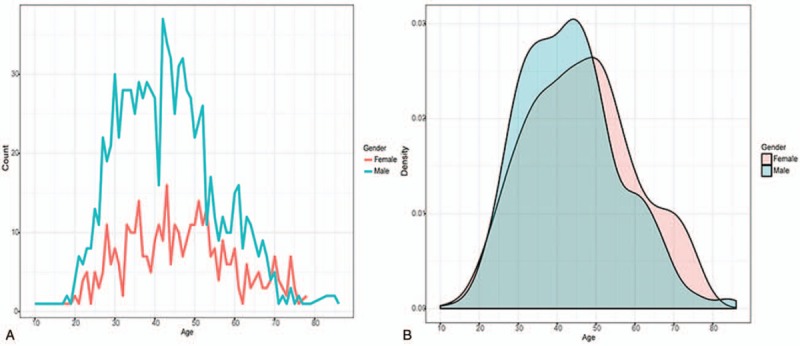
Gender/age distribution. (A) Frequency distribution. (B) Density distribution.

In the present study, 1357 patients participated in this study, of which 969 were males (71.4%) and 388 were females (28.6%). The general information of our participators was shown in Table 1. Specific age and gender distributions were shown in Figure 1. The male and female patients had a similar distribution pattern, both reaching the peak value in the middle age. Combined with gender, the 14 indexes kept for subsequent analysis were listed as serum albumin (ALB; 43.16 ± 5.93), alkaline phosphatase (ALP; 127.60 ± 59.37), alanine aminotransferase (ALT; 96.15 ± 167.70), aspartate aminotransferase (AST; 63.32 ± 107.73), direct bilirubin (DB; 7.54 ± 12.84), indirect bilirubin (IB; 19.25 ± 17.29), Gamma-glutamyltransferase (GGT; 71.17 ± 120.66), serum globulin (GLB; 28.18 ± 4.83), platelet (PLT; 160.05 ± 112.93), AST to platelet ratio index (APRI; 1.67 ± 3.51), AST-to-ALT ratio index (AARI; 0.89 ± 0.56), fibrosis index based on the 4 factor (FIB-4; 3.35 ± 8.87), controlled attenuation parameter (CAP; 228.43 ± 29.88), and liver stiffness measurement (LSM; 12.78 ± 12.02) separately. The detail values of each biological parameter were shown in Table 2.

**Table 1:**
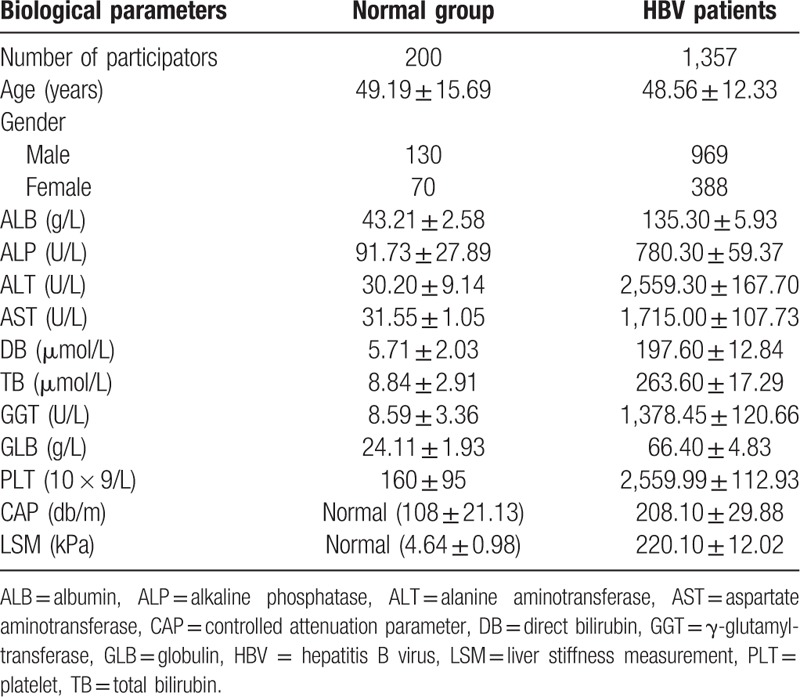
The general information of our participators.

**Table 2:**
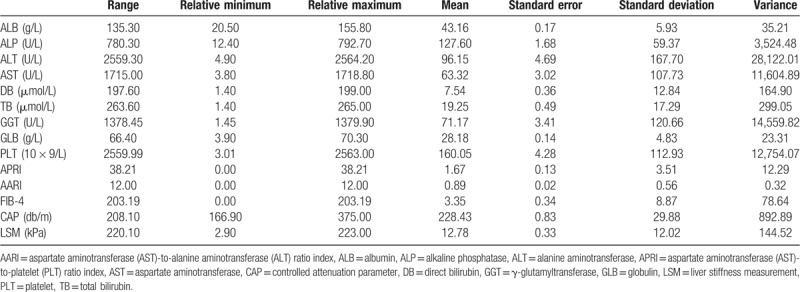
Random distribution of the 14 indexes.

### 3.2 Analysis of communalities and correlations among the 14 clinical indices

**Figure 2:**
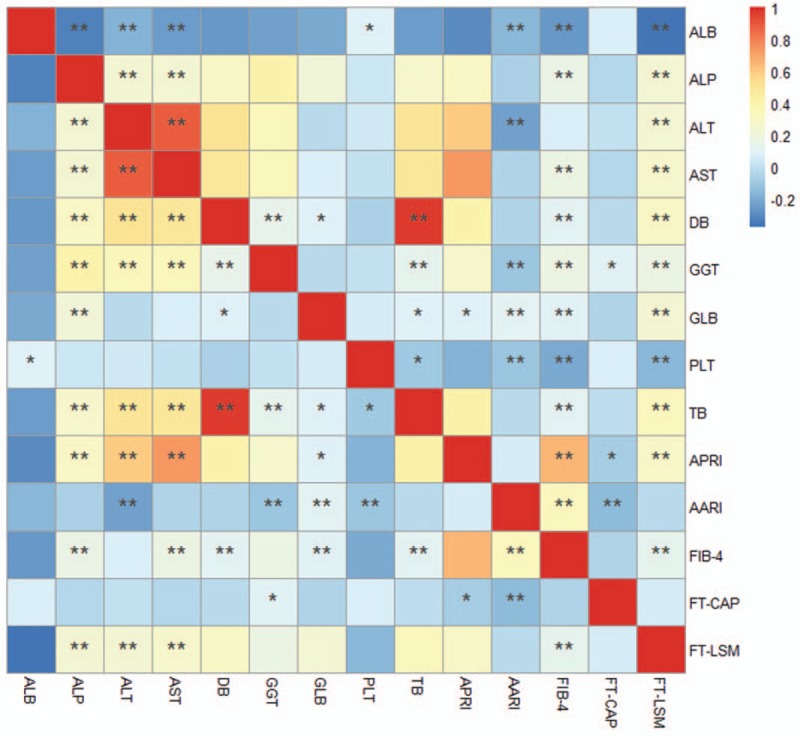
Analysis of correlations among the 14 indices. ∗∗ *P* ≤ .01; ∗*P* ≤ .05; changing color from red to blue represents the magnitude of the coefficient of correlation among variables.

We subjected the 14 clinical indices to correlation analyses with SPSS and constructed the heatmap by R scripts (Fig. 2). TB and DB were significantly and strongly correlated (*r* = 0.897, *P* = .000), as were ALT and AST (*r* = 0.880, *P* = .000). GGT and ALP were moderately correlated (*r* = 0.447, *P* = .000). DB (*r* = 0.515, *P* = .000), TB (*r* = 0.486, *P* = .000), and ALT were moderately correlated. DB (*r* = 0.489, *P* = .000), TB (*r* = 0.475, *P* = .000), and AST were moderately correlated. The remaining indexes were extremely weakly correlated or uncorrelated.

Furthermore, factor analysis was applied to compute the communalities of the 14 clinical indexes (Table 3). The communalities of various indexes were ranked as follows: TB (0.894) > DB (0.882) > APRI (0.856) > ALT (0.849) > AST (0.839) > FIB-4 (0.736) > GGT (0.641) > PLT (0.636) > GLB (0.564) > ALP (0.561) > Fibrotouch-LSM (0.559) > AARI (0.554) > ALB (0.537) > Fibrotouch-CAP (0.524). The commonality of each of the 14 indices was >0.5 (the correlation was considered strong when the theoretical numerical value >0.4). There were five principal components with a characteristic value >1 in the matrix. The rate of cumulative contribution to the total variance was 68.81%. The orthogonal rotation method with Kaiser Normalization was used to establish a factor load matrix. The first factors included AST, APRI, ALT, GGT, and PLT. The rate of cumulative contribution to the total variance was 29.75%. Secondary factors were TB and DB. The cumulative contribution to the total variance was 12.63%. The tertiary factors were ALP, GLB, and LSM. The cumulative contribution to the total variance was 9.54%. The quaternary factors were AARI and FIB-4. The cumulative contribution to the total variance was 9.28%. The quinary factors were CAP and ALB. The cumulative contribution to the total variance was 7.60%.

**Table 3:**
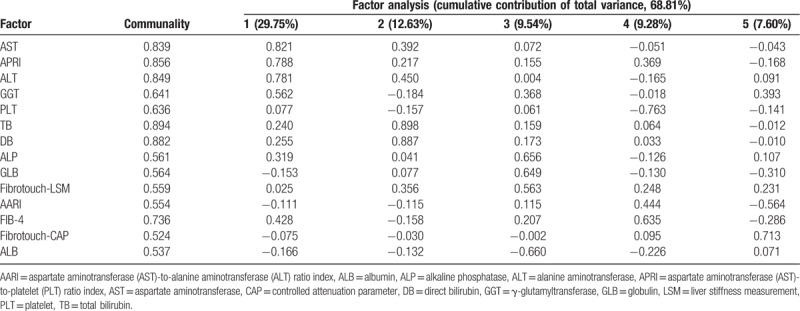
Statistical results of the communalities of the 14 indexes of the CHB patients.

### 3.3 Impact of age and gender on CAP and LSM

The specific results of CAP or LSM were grouped by gender and age separately. For instance, CAP and LSM were both grouped into male or female by gender, while both CAP and LSM were divided into youth, mid-aged, and the elder by age. The detail information was shown in Table 4.

**Table 4:**
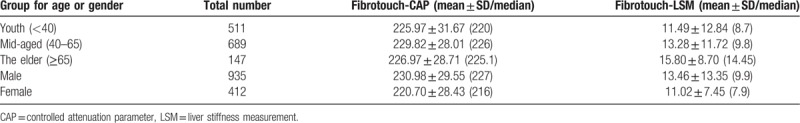
Distribution of groups by age and gender within the two indices of Fibrotouch measurement.

**Figure 3:**
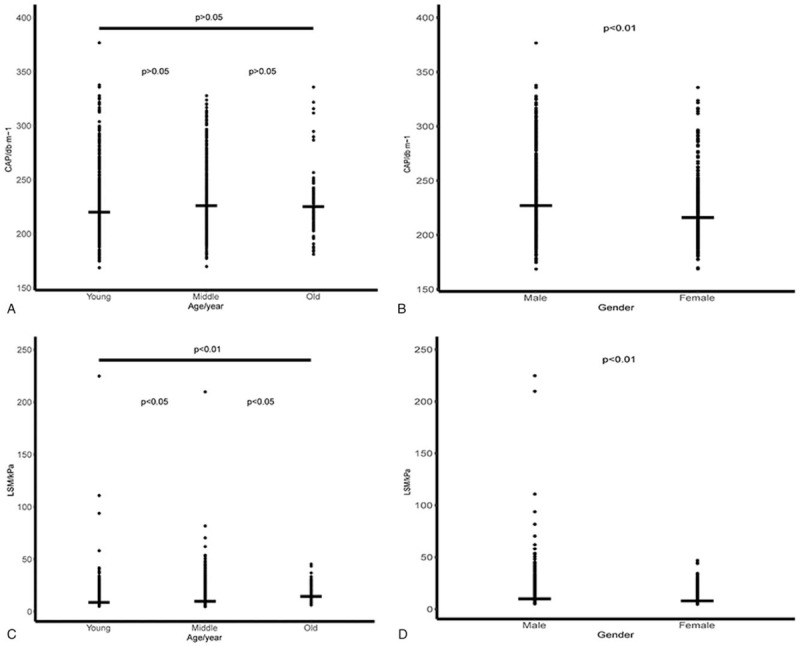
Scatter diagram of the two Fibrotouch measurement indices in the gender and age groups. (A) Statistical analysis of CAP parameter by age. (B) Statistical analysis of CAP parameter by gender. (C) Statistical analysis of LSM parameter by age. (D) Statistical analysis of LSM parameter by gender. The transverse line represents the median value within the group; the differences among various groups are considered significant when *P* ≤ .05 and were highly significant when *P* ≤ .01.

To evaluate the effects of age and gender on the Fibrotouch measurement, we plotted to scatter diagrams for various groups by age and gender, while statistically significant differences were also calculated (Fig. 3). Groups categorized by age did not have significant effects on CAP parameters, while gender had a significant effect on the CAP indexes. Specifically, CAP values of the male patients were significantly higher than those of the female patients. LSM values in male patients were significantly higher than those of female patients. Moreover, the LSM value of the elderly group was significantly higher than that of the young and middle-age groups, while the value of the middle-age group was significantly larger than that of the young group. Collectively, the result revealed that the gender and age groups had significant effects on the LSM indexes.

### 3.4 Factors associated with CAP

According to the Fibrotouch instrument indexes, CAP were divided into four groups based on estimated values: normal (≤240); mild (240–265), moderate (265–295); and severe (>295)^[17]^ (Table 5). The random statistical result showed that CAP value of most HBV patients was within the normal standard range. Only a very small number of patients were within the severe range, which indicated the occurrence of severe fat lesions in liver cells or in other words, the patients had severe fatty liver disease but had no medical history of fatty liver.

**Table 5:**
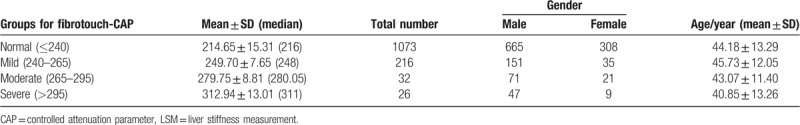
Basic information of various groups by CAP indexes.

**Figure 4:**
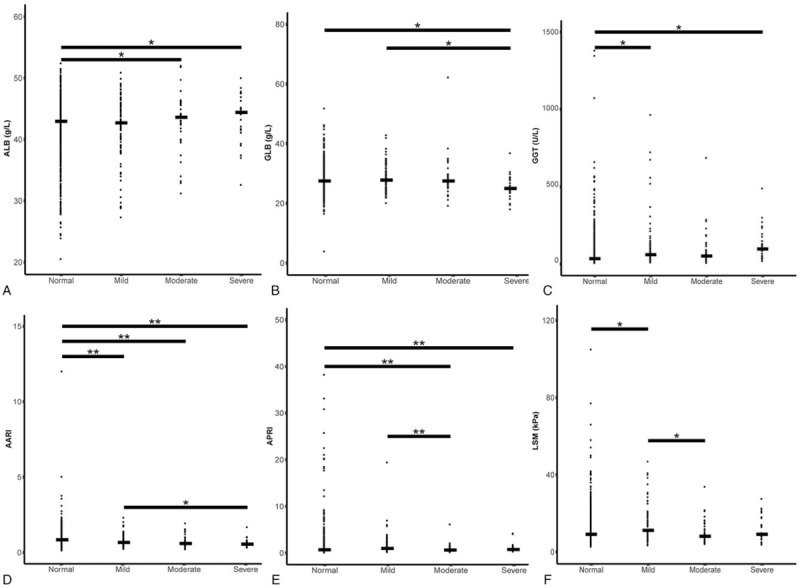
Distribution scatter diagram of CAP groups among various measuring indices. (A) Statistical analysis of ALB parameter among CAP groups. (B) Statistical analysis of GLB parameter among CAP groups. (C) Statistical analysis of GGT parameter among CAP groups. (D) Statistical analysis of AARI parameter among CAP groups. (E) Statistical analysis of APRI parameter among CAP groups. (F) Statistical analysis of LSM parameter among CAP groups. Only the indices with significant inter-group differences were retained; the transverse line represents the median value within a group; ∗*P* ≤ .05, indicating the inter-group differences are significant; ∗∗*P* ≤ .01, indicating the inter-group differences were highly significant.

Statistical analysis was conducted on the significant differences in the other 13 indexes among the four groups (Fig. 4). The statistical analysis found that the four groups had significant differences in the six factors: ALB, GLB, GGT, AARI, APRI, and LSM. ALB concentrations in the normal group were significantly lower than that of the moderate and severe groups, indicating that more severe lesions in the hepatocyte fat led to lower levels of synthesized ALB. GLB concentrations in the normal and mild groups were significantly lower than those of the severe group, suggesting that a higher level of hepatocyte fat led to lower GLB synthesis. AARI in normal group was significantly lower than that of the mild, moderate, and severe groups. The index in the mild group was significantly lower than that of the severe group, suggesting that a high level of hepatocyte fat led to a higher AARI ratio. There were no linear relationships in differences in GGT, APRI, and LSM among the four groups.

### 3.5 Factors associated with LSM

**Figure 5:**
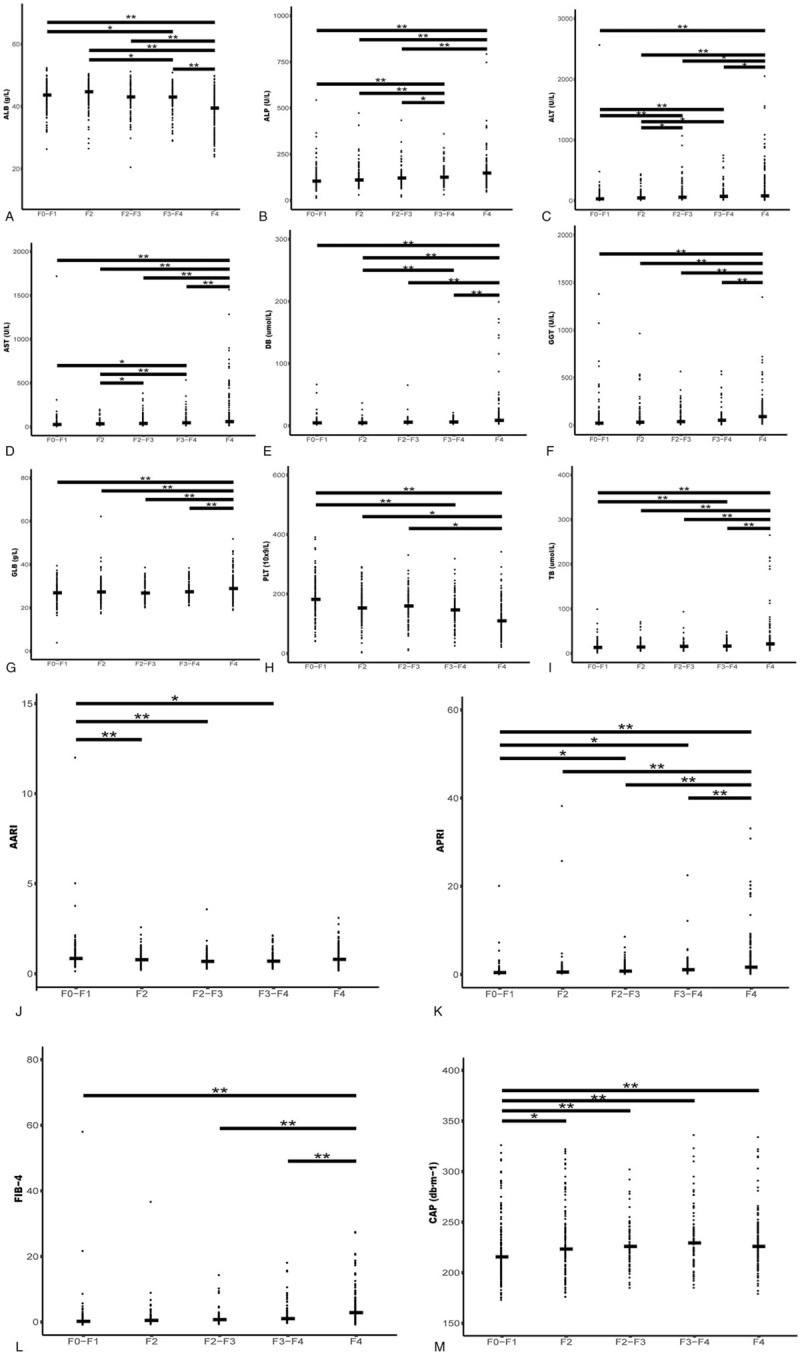
Distribution scatter diagrams of LSM groups for various measuring indices. (A) Statistical analysis of ALB parameter among LSM groups. (B) Statistical analysis of ALP parameter among LSM groups. (C) Statistical analysis of ALT parameter among LSM groups. (D) Statistical analysis of AST parameter among LSM groups. (E) Statistical analysis of DB parameter among LSM groups. (F) Statistical analysis of GGT parameter among LSM groups. (G) Statistical analysis of GLB parameter among LSM groups. (H) Statistical analysis of PLT parameter among LSM groups. (I) Statistical analysis of TB parameter among LSM groups. (J) Statistical analysis of AARI parameter among LSM groups. (K) Statistical analysis of APRI parameter among LSM groups. (L) Statistical analysis of FIB-4 parameter among LSM groups. (M) Statistical analysis of CAP parameter among LSM groups. Only the indices with significant inter-group differences were retained; the transverse line represents the median value within a group; ∗*P* ≤ .05, indicating the inter-group differences are significant; ∗∗*P* ≤ .01, indicating the inter-group differences are highly significant.

According to the Fibrotouch instrument indexes, the LSM was divided into five groups: F0-F1, F2, F2–F3, F3–F4, and F4^[21]^ (Table 6). There were significant differences in 13 factors (ALB, ALP, ALT, AST, DB, GGT, GLB, PLT, TB, AARI, APRI, FIB-4, and CAP) among the five groups (Fig. 5, A and B). Furthermore, there was a good linear relationship among eight factors: ALP, ALT, GGT, GLB, PLT, TB, APRI, and CAP. The ALP values of the first three groups were significantly lower than those of the F3–F4 and F4 groups, suggesting that the ALP concentrations in the latter two groups divided by LSM were significantly different from those in the first three groups. ALT values in each of the first four groups were significantly lower than those of the F4 group, and the values of the first two groups were significantly lower than those in the F3–F4 and F2–F3 groups, suggesting that ALB could be roughly divided into three ranges (F0–F2, F2–F4, and F4) with significant differences. In terms of GGT and GLB, the values in the first four groups were extremely significantly lower than those of the F4 group. The two indexes could be roughly divided into two ranges, F0–F4 and F4, with significant differences. In terms of PLT, the values in the first three groups were significantly higher than those in the F4 group. Values in the F0–F1 groups were significantly higher than those of the F3–F4 groups, suggesting that a higher level of hepatic fibrosis led to relatively lower PLT content. In contrast, in terms of TB, the values in the first three groups were significantly lower than those of the F4 group. The values in the F0–F1 groups were significantly lower than those of the F3–F4 group, suggesting that higher levels of hepatic fibrosis led to higher levels of TB. In terms of APRI, the values in the first four groups were much lower than those of the F4 group. Values in F0–F1 were significantly lower than those of the F2–F3 and F3–F4 groups, suggesting that higher levels of fibrosis led to larger APRI values. CAP values in the F0–F1 group were significantly lower than those of the remaining four groups, suggesting that LSM parameters could be roughly divided into two ranges, F0–F1 and F2–F4, within the numerical range of CAP.

**Table 6:**
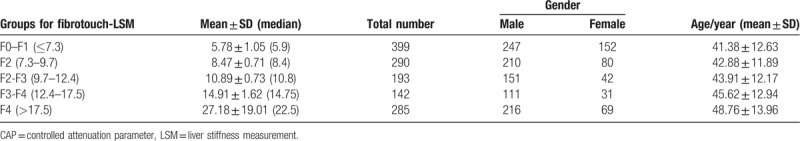
Basic information of the various groups grouped by LSM index.

### 3.6 Principal component analysis of all people and a significant difference of LSM and CAP by age and gender among healthy control

**Figure 6:**
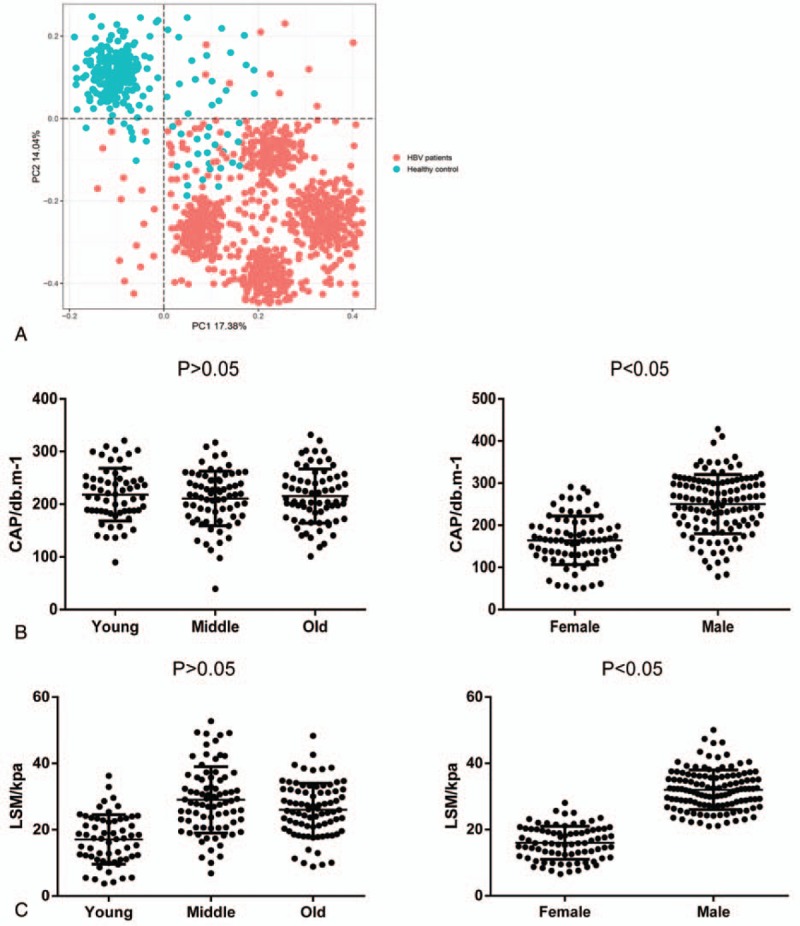
PCA analysis and correlation analysis of healthy control on CAP and LSM. (A) PCA analysis of HBV patients and healthy control. (B) Correlation analysis between age or gender and CAP value in healthy control. (C) Correlation analysis between age or gender and LSM value in healthy control.

In this study, we focused on the physiochemical or clinical differences within HBV patients and showed a significant impact on fibrotouch detection by age and gender. To make further validation, we gathered 200 healthy volunteers to receive fibrotouch detection whose other indicators were all normally standard. We first applied the PCA analysis to detect the different clustering pattern between HBV patients and healthy control (Fig. 6A). The results showed that specimens from a healthy control were obviously clustering tightly together, while the specimens from the HBV patients were clustering into several subgroups. In contrast, we divided the healthy control into several subgroups based on gender or age and conducted the correlated analysis with CAP and LSM (Fig. 6B and C). The results showed that gender significantly impacted the CAP and LSM values, while age had barely difference between CAP and LSM distribution.

## 4 Discussion

Over the decades, the transient elastography has been widely applied in clinical diagnosis because of advantages including non-invasiveness, rapidness, simplicity, and high patient compliance. The usefulness of LSM in determining liver fibrosis has been established, while the detection accuracy may be influenced by various factors. For instance, Ji et al showed the significance of the applicability of LSM on the assessment of hepatitis B related fibrosis and cirrhosis.^[22]^ Jia et al also showed that compared with current serum biomarkers, transient elastography acts as a reliable noninvasive technique to predict significant liver fibrosis in Chinese patients with CHB, while abnormal inflammatory activity could lead to elevated stiffness values unrelated to histological fibrosis stage.^[23]^

In the present study, we focused on the correlations of measurement indexes of the HBV patients within a large data size, the result showed that gender had significant effects on the Fibrotouch measurement among the random statistics of HBV patients (*P* ≤ .05), which is consistent with the previous study.^[24]^ The previous study pointed out that age does not affect the LSM value on fibroscan,^[25]^ while up to date, there existed few studies on the possible and potential effects of age in Fibrotouch detection. Interestingly, our research indicated the similar result that the age factor had no obvious effect on the hepatic fibrosis parameter LSM and CAP (*P* > .05) considering both in HBV patients and the healthy control. In other words, age did barely any differences on Fibrotouch. Collectively, our results suggested that the effect of gender needs to be accounted for in the Fibrotouch measurement, while the age parameter could be ignored.

Ding et al found that the value of LSM was significantly correlated with ALT, ALP, cholinesterase (ChE), TB, DB, and IB.^[26]^ Similar with previous result, we found that there existed significant linear differences of the eight factors (ALP, ALT, GGT, GLB, PLT, TB, APRI, and CAP) among the various groups, which showed that the degree of abnormal values of these indexes was frequently associated with the severity of hepatitis. These findings indicated that evaluation of combined LSM value with these serums may enhance the accuracy of CHB assessment.

According to the previous experience, more severe lesions in the hepatocyte fat would lead to lower levels of synthesized ALB, while a higher level of hepatocyte fat led to lower GLB synthesis. Moreover, a high level of hepatocyte fat often led to higher AARI. Our result also showed that abnormal values of the four indexes (TB, DB, AST, and ALT) were generally occurred along with the definite HBV diagnosis. The results indicated a huge clinical potential of these parameters in HBV auxiliary screening.

Collectively, our research focused on the correlation analysis of multiple indexes including routine serum indexes and Fibrotouch measurements in a large cohort of CHB patients. Our results showed that in CHB patients, several conventional indexes like ALT, ALP, and GGT have obvious correlation with the incidence or severity of CHB. Besides, we found several serum indexes frequently kept pace with the results of Fibrotouch measurements, which indicated the effective application of combination. Moreover, we proposed that gender difference often significantly affected the Fibrotouch measurement especially in LSM detection. To eliminate the significant difference of LSM value affected by gender, two different kinds of standards in dealing with males and females should be set in the future.

However, there still exists limitation in our present study. First, the difference of LSM value may be affected by different physiological/pathological condition. Secondly, considering the comprehensive complication, in the future research, we will include a group of patients with hepatitis B complicated with fatty liver disease and the group of patients with fatty liver disease in our subsequent collection and analyses of cases, which will help to clarify the principal factors among the various diseases.

## Author contributions

**Data curation:** Zhonghua Li.

**Formal analysis:** Yinhua Gong.

**Project administration:** Yonglan Pu.

**Software:** Xiaofang Zhu.

**Writing – original draft:** Jie Wang.
